# Supplementary material for: Factors Influencing Self-Reported Medication Use in the Survey of Health Aging and Retirement in Europe (SHARE) Dataset
Source: Healthcare (Basel). 2021 Dec 18;9(12):1752. doi: 10.3390/healthcare9121752 (PMC8701040; doi:10.3390/healthcare9121752)
Supplement: Supplementary file 1 [file healthcare-09-01752-s001.zip › healthcare-1502725-supplementary.pdf]

Supplement

Supplement Table S1. Univariate Spearman correlations between self-report of disorders/conditions and self-report of medication use in people with impaired (ten words list learning delayed recall total < 5 correct) and unimpaired memory 2

Supplement Table S2. Binomial Logistic Regression: Drugs for: diabetes 3

Supplement Table S3. Binomial Logistic Regression: Drugs for: chronic bronchitis 4

Supplement Table S4. Binomial Logistic Regression: Drugs for: high blood cholesterol 5

Supplement Table S5. Binomial Logistic Regression: Drugs for: anxiety or depression 5

Supplement Table S6. Binomial Logistic Regression: Drugs for: joint pain 6

Supplement Table S7. Binomial Logistic Regression: Drugs for: stomach burns 6

Supplement Table S8. Binomial Logistic Regression: Drugs for: sleep problems..... 7

Supplement Table S9. Binomial Logistic Regression: Drugs for: coronary diseases 7

Supplement Table S10. Binomial Logistic Regression: Drugs for: other heart diseases 8

Supplement Table S11. Binomial Logistic Regression: Drugs for: other pain 8

Supplement Table S12. Binomial Logistic Regression: Drugs for: osteoporosis 9

Supplement Table S13. Binomial Logistic Regression: Drugs for: suppressing inflammation (only glucocorticoids or steroids) 9

Supplement Table S14. Binomial Logistic Regression: Drugs for: other 10

Supplement Table S15. Binominal Logistic regression: Drug for: Hypertension (after exclusion of people with Alzheimer´s disease, dementia, senility)..... 10

Supplement Table S16. Mediation model for Drugs for: diabetes..... 11

Supplement Table S1. Univariate Spearman correlations between self-report of disorders/conditions and self-report of medication use in people with impaired (ten words list learning delayed recall total < 5 correct) and unimpaired memory

	Drugs selected for													
**Ever diagnosed/ currently having: Impaired memory vs Unimpaired memory***	**High blood cholesterol**	**high blood pressure**	**coronary diseases**	**other heart diseases**	**diabetes**	**chronic bronchitis**	**anxiety or depression**	**joint pain**	**stomach burns**	**high blood cholesterol**	**sleep problems**	**osteoporosis**	**suppressing inflammation**	**none**
**High blood cholesterol**	**.709**	.238	.135	.109	.169	.040	.069	.087	.112	.056	.089	.070	.026	-.219
	**.688**	.232	.151	.118	.149	.042	.059	.086	.111	.066	.089	.056	.027	-.263
**High blood pressure or hypertension**	.244	**.807**	.144	.156	.181	.049	.052	.119	.083	.080	.081	.045	.037	-.399
	.250	**.860**	.143	.143	.184	.032	.033	.114	.072	.059	.057	.027	.021	-.469
**Heart attack**	.165	.159	**.420**	**.507**	.091	.060	.042	.085	.079	.060	.085	.033	.036	-.171
	.196	.169	**.463**	**.509**	.106	.063	.035	.091	.072	.062	.079	.024	.049	-.182
**Diabetes or high blood sugar**	.216	.202	.113	.086	**.884**	.038	.051	.079	.067	.043	.064	.009*	.023	-.186
	.209	.206	.125	.095	**.887**	.044	.028	.076	.049	.043	.049	.012*	.020	-.198
**Chronic lung disease**	.045	.056	.093	.090	.042	**.519**	.055	.091	.091	.074	.077	.056	.075	-.080
	.043	.054	.073	.086	.041	**.532**	.070	.099	.087	.064	.080	.074	.096	-.106
**Other affective/ emotional disorders**	.051	.037	.052	.057	.033	.060	**.540**	.109	.113	.130	.251	.076	.062	-.094
	.042	.032	.041	.052	.017	.077	**.515**	.108	.107	.096	.240	.056	.052	-.114
**Rheumatoid arthritis**	.063	.099	.060	.086	.059	.073	.090	**.354**	.108	.168	.108	.155	.120	-.120
	.054	.095	.070	.078	.053	.064	.060	**.352**	.087	.144	.086	.121	.133	-.136
	.076	.083	.061	.069	.036	.067	.084	**.338**	.134	.175	.120	.240	.107	-.136

**Osteoarthritis/ other rheumatism**	.074	.083	.049	.059	.029	.064	.076	**.332**	.138	.151	.116	.171	.099	-.156
**Stomach or duodenal ulcer, peptic ulcer**	.050<br>.051	.046<br>.052	.059<br>.067	.065<br>.062	.026<br>.036	.070<br>.077	.077<br>.080	.106<br>.092	**.328**<br>**.320**	.107<br>.106	.090<br>.087	.074<br>.063	.066<br>.053	-.064<br>-.087
**Alzheimer's disease, dementia, senility**	.032<br>.029	.024<br>.034	.068<br>.073	.046<br>.021	.035<br>.031	.036<br>.041	.133<br>.072	.050<br>.044	.028<br>.046	.052<br>.042	.093<br>.072	.039<br>.025	.031<br>.040	-.058<br>-.013*
**Stroke**	.103<br>.101	.103<br>.102	.125<br>.298	.297<br>.116	.142<br>.058	.074<br>.043	.052<br>.061	.066<br>.058	.060<br>.063	.056<br>.065	.063<br>.067	.087<br>.032	.042<br>.037	.033<br>-.090

*note: first line: impaired memory (N=26752), second line unimpaired memory (N=47703)

for all p< 0.001 except *p = 0.028

Supplement Table S2. Binomial Logistic Regression: Drugs for: diabetes

Predictor	Estimate	p	Odds ratio	95% CI Lower	95% CI Upper	Model Fit Measures		Overall Model Test		
						R^2_N	AIC	χ^2	df	p
**Model 1**										
Intercept	-0.4194	< .001	0.657	0.657	0.757	0.108	9551	751	3	< .001
Depression	-0.0165	0.145	0.984	0.984	1.006					
Memory	-0.0950	< .001	0.909	0.909	0.933					
Polypharmacy No – Yes	-1.3233	< .001	0.266	0.239	0.296					
**Model 2**										
Intercept	-2.98091	< .001	0.0507	0.0143	0.180	0.828	2599	7714	8	< .001
Depression	-0.01520	0.580	0.9849	0.9333	1.039					
Memory	-0.05247	0.088	0.9489	0.8933	1.008					
Polypharmacy No - Yes	-1.02490	< .001	0.3588	0.2759	0.467					
Age	0.00184	0.817	1.0018	0.9864	1.018					
Number of chronic Illnesses	-0.19788	< .001	0.8205	0.7550	0.892					
Instrumental activities of daily life	-0.04209	0.278	0.9588	0.8886	1.034					
Sex: Female – Male	-0.06709	0.575	0.9351	0.7398	1.182					
Diabetes: Selected – Not selected	6.62441	< .001	753.2572	574.8532	987.028					
**Model comparison: 1-2**								6962	5	< .001
Note. Estimates represent the log odds of "Drugs for: diabetes = Selected" vs. "Drugs for: diabetes = Not selected"										

Supplement Table S3. Binomial Logistic Regression: Drugs for: chronic bronchitis

Predictor	Estimate	p	Odds ratio	95% CI Lower	95% CI Upper	Model Fit Measures		Overall Model Test		
						R^2_N	AIC	χ^2	df	p
**Model 1**										
Intercept	-2.9829	< .001	0.657	0.0506	0.0668	0.0535	3403	192	3	< .001
Depression	0.0887	< .001	0.984	1.0928	1.1374					
Memory	0.0418	0.087	0.909	1.0427	1.0937					
Polypharmacy No – Yes	-1.2295	< .001	0.266	0.2924	0.3608					
**Model 2**										
Intercept	-4.98673	< .001	0.00683	0.00180	0.0259	0.4878	1998	1607	8	< .001
Depression	0.02920	0.299	1.02963	0.97445	1.0879					
Memory	0.01945	0.542	1.01964	0.95783	1.0854					
Polypharmacy No - Yes	-0.61019	< .001	0.54325	0.41248	0.7155					
Age	0.00104	0.900	1.00104	0.98493	1.0174					
Number of chronic Illnesses	0.05479	0.158	1.05632	0.97896	1.1398					
Instrumental activities of daily life	-0.01273	0.743	0.98735	0.91492	1.0655					
Sex: Female – Male	0.18944	0.138	1.20857	0.94095	1.5523					
Chronic Bronchitis: Selected – Not selected	4.37785	< .001	79.66686	60.13950	105.5348					
**Model comparison: 1-2**								1415	5	< .001
Note. Estimates represent the log odds of "Drugs for: chronic bronchitis = Selected" vs. "Drugs for: chronic bronchitis = Not selected"										

Supplement Table S4. Binomial Logistic Regression: Drugs for: high blood cholesterol

Predictor	Estimate	p	Odds ratio	95% CI Lower	95% CI Upper	Model Fit Measures		Overall Model Test		
						R^2_N	AIC	χ^2	df	p
**Model 1**										
Intercept	0.4047	< .001	1.499	1.334	1.684	0.0699	14299	590	3	< .001
Depression	-0.0431	< .001	0.958	0.941	0.975					
Memory	-0.0203	0.037	0.980	0.961	0.999					
Polypharmacy No – Yes	-1.0407	< .001	0.353	0.324	0.385					
**Model 2**										
Intercept	-1.4306	< .001	0.239	0.126	0.454	0.6331	7890	7009	8	< .001
Depression	-0.0520	< .001	0.949	0.923	0.976					
Memory	-0.0138	0.372	0.986	0.957	1.017					
Polypharmacy No - Yes	-1.3191	< .001	0.267	0.232	0.308					
Age	0.0130	0.001	1.013	1.005	1.021					
Number of chronic Illnesses	-0.0836	< .001	0.920	0.877	0.964					
Instrumental activities of daily life	-0.0858	< .001	0.918	0.879	0.959					
Sex: Female – Male	-0.4280	< .001	0.652	0.578	0.735					
High blood cholesterol: Selected – Not selected	4.1551	< .001	63.758	55.266	73.557					
**Model comparison: 1-2**								6419	5	< .001
Note. Estimates represent the log odds of "Drugs for: high blood cholesterol = Selected" vs. "Drugs for: high blood cholesterol = Not selected"										

Supplement Table S5. Binomial Logistic Regression: Drugs for: anxiety or depression

Predictor	Estimate	p	Odds ratio	95% CI Lower	95% CI Upper	Model Fit Measures		Overall Model Test		
						R^2_N	AIC	χ^2	df	p
**Model 1**										
Intercept	-2.9394	< .001	0.0529	0.0432	0.0648	0.156	5902	814	3	< .001
Depression	0.2927	< .001	1.3401	1.3043	1.3768					
Memory	0.0372	0.028	1.0379	1.0041	1.0728					
Polypharmacy No – Yes	-0.8914	< .001	0.4101	0.3559	0.4725					
**Model 2**										
Intercept	-0.2837	0.522	0.753	0.316	1.795	0.400	4503	2222	8	< .001
Depression	0.1605	< .001	1.174	1.134	1.215					
Memory	-0.0188	0.385	0.981	0.941	1.024					
Polypharmacy No - Yes	-1.1189	< .001	0.327	0.271	0.394					
Age	-0.0354	< .001	0.965	0.955	0.976					
Number of chronic Illnesses	-0.1544	< .001	0.857	0.810	0.906					
Instrumental activities of daily life	0.1329	< .001	1.142	1.090	1.196					
Sex: Female – Male	0.5091	< .001	1.664	1.389	1.992					
Anxiety or depression: Selected – Not selected	3.4521	< .001	31.567	25.723	38.738					
**Model comparison: 1-2**								1408	5	< .001

Note. Estimates represent the log odds of "Drugs for: anxiety or depression = Selected" vs. "Drugs for: anxiety or depression = Not selected"

Supplement Table S6. Binomial Logistic Regression: Drugs for: joint pain

**Predictor**	**Estimate**	**p**	**Odds ratio**	**95% CI Lower**	**95% CI Upper**	**Model Fit Measures**		**Overall Model Test**		
						**R^2_N**	**AIC**	**χ^2**	**df**	**p**
**Model 1**										
Intercept	-0.8145	< .001	0.443	0.390	0.503	0.117	11506	913	3	< .001
Depression	0.1411	< .001	1.151	1.130	1.174					
Memory	-0.0291	0.010	0.971	0.950	0.993					
Polypharmacy No – Yes	-0.9917	< .001	0.371	0.338	0.407					
**Model 2**										
Intercept	-1.23126	< .001	0.292	0.169	0.504	0.324	9764	2741	9	< .001
Depression	0.06074	< .001	1.063	1.038	1.087					
Memory	-0.04150	0.002	0.959	0.934	0.985					
Polypharmacy No - Yes	-0.77888	< .001	0.459	0.408	0.516					
Age	-0.00902	0.008	0.991	0.984	0.998					
Number of chronic Illnesses	0.04104	0.043	1.042	1.001	1.084					
Instrumental activities of daily life	0.07923	< .001	1.082	1.047	1.119					
Sex: Female – Male	0.39488	< .001	1.484	1.331	1.655					
Rheumatoid arthritis: Selected – Not selected	1.73568	< .001	5.673	4.913	6.549					
Osteoarthritis: Selected – Not selected	1.47294	< .001	4.362	3.906	4.872					
**Model comparison: 1-2**								1828	6	< .001
Note. Estimates represent the log odds of "Drugs for: joint pain = Selected" vs. "Drugs for: joint pain = Not selected"										

Supplement Table S7. Binomial Logistic Regression: Drugs for: stomach burns

**Predictor**	**Estimate**	**p**	**Odds ratio**	**95% CI Lower**	**95% CI Upper**	**Model Fit Measures**		**Overall Model Test**		
						**R^2_N**	**AIC**	**χ^2**	**df**	**p**
**Model 1**										
Intercept	-1.7283	< .001	0.178	0.152	0.208	0.0692	8378	429	3	< .001
Depression	0.1029	< .001	1.108	1.083	1.134					
Memory	0.0394	0.004	1.040	1.012	1.069					
Polypharmacy No – Yes	-0.9573	< .001	0.384	0.342	0.431					
**Model 2**										
Intercept	-2.2739	< .001	0.103	0.0540	0.196	0.1652	7763	1055	8	< .001
Depression	0.0771	< .001	1.080	1.0518	1.109					
Memory	0.0415	0.008	1.042	1.0107	1.075					
Polypharmacy No - Yes	-0.7796	< .001	0.459	0.4003	0.525					
Age	1.59e-4	0.969	1.000	0.9923	1.008					

Number of chronic Illnesses	0.1044	< .001	1.110	1.0652	1.157					
Instrumental activities of daily life	-0.0204	0.307	0.980	0.9421	1.019					
Sex: Female – Male	0.0919	0.143	1.096	0.9692	1.240					
Stomach burns: Selected – Not selected	2.3843	< .001	10.852	8.7866	13.402					
**Model comparison: 1-2**								625	5	< .001
Note. Estimates represent the log odds of "Drugs for: stomach burns = Selected" vs. "Drugs for: stomach burns = Not selected"										

Supplement Table S8. Binomial Logistic Regression: Drugs for: sleep problems

**Predictor**	**Estimate**	**p**	**Odds ratio**	**95% CI Lower**	**95% CI Upper**	**Model Fit Measures**		**Overall Model Test**		
						**R^2_N**	**AIC**	**χ^2**	**df**	**p**
**Model 1**										
Intercept	-2.27358	< .001	0.103	0.0861	0.123	0.154	6828	891	3	< .001
Depression	0.24425	< .001	1.277	1.2452	1.309					
Memory	-0.00945	0.544	0.991	0.9608	1.021					
Polypharmacy No – Yes	-1.04324	< .001	0.352	0.3096	0.401					
**Model 2**										
Intercept	-3.9033	< .001	0.0202	0.0101	0.0402	0.171	6733	994	7	< .001
Depression	0.2079	< .001	1.2311	1.1985	1.2646					
Memory	0.0111	0.515	1.0112	0.9779	1.0455					
Polypharmacy No - Yes	-0.9356	< .001	0.3923	0.3395	0.4534					
Age	0.0144	< .001	1.0145	1.0061	1.0229					
Number of chronic Illnesses	0.0483	0.021	1.0495	1.0072	1.0935					
Instrumental activities of daily life	0.0435	0.017	1.0444	1.0077	1.0825					
Sex: Female – Male	0.5880	< .001	1.8003	1.5629	2.0738					
**Model comparison: 1-2**								103	4	< .001
Note. Estimates represent the log odds of "Drugs for: stomach burns = Selected" vs. "Drugs for: stomach burns = Not selected"										

Supplement Table S9. Binomial Logistic Regression: Drugs for: coronary diseases

**Predictor**	**Estimate**	**p**	**Odds ratio**	**95% CI Lower**	**95% CI Upper**	**Model Fit Measures**		**Overall Model Test**		
						**R^2_N**	**AIC**	**χ^2**	**df**	**p**
**Model 1**										
Intercept	-0.7795	< .001	0.459	0.394	0.534	0.144	8133	932	3	< .001
Depression	0.0464	< .001	1.048	1.023	1.072					
Memory	-0.0998	< .001	0.905	0.880	0.931					
Polypharmacy No – Yes	-1.5129	< .001	0.220	0.196	0.248					
**Model 2**										
Intercept	-3.66471	< .001	0.0256	0.0135	0.0485	0.197	7780	1293	7	< .001
Depression	0.04391	0.001	1.0449	1.0177	1.0728					
Memory	-0.03497	0.027	0.9656	0.9362	0.9960					
Polypharmacy No - Yes	-1.14306	< .001	0.3188	0.2799	0.3632					
Age	0.02960	< .001	1.0300	1.0221	1.0380					

Number of chronic Illnesses	0.23762	< .001	1.2682	1.2211	1.3172					
Instrumental activities of daily life	0.00863	0.623	1.0087	0.9746	1.0439					
Sex: Female – Male	-0.72569	< .001	0.4840	0.4295	0.5454					
**Model comparison: 1-2**								361	4	< .001
Note. Estimates represent the log odds of "Drugs for: coronary diseases = Selected" vs. "Drugs for: coronary diseases = Not selected"										

Supplement Table S10. Binomial Logistic Regression: Drugs for: other heart diseases

**Predictor**	**Estimate**	**p**	**Odds ratio**	**95% CI Lower**	**95% CI Upper**	**Model Fit Measures**		**Overall Model Test**		
						**R^2_N**	**AIC**	**χ^2**	**df**	**p**
**Model 1**										
Intercept	-0.9114	< .001	0.402	0.347	0.466	0.121	8696	801	3	< .001
Depression	0.0458	< .001	1.047	1.023	1.071					
Memory	-0.0401	0.003	0.961	0.936	0.987					
Polypharmacy No – Yes	-1.4176	< .001	0.242	0.216	0.271					
**Model 2**										
Intercept	-3.70606	< .001	0.0246	0.0134	0.0451	0.155	8470	1035	7	< .001
Depression	0.03817	0.003	1.0389	1.0130	1.0655					
Memory	0.01855	0.213	1.0187	0.9894	1.0489					
Polypharmacy No - Yes	-1.10724	< .001	0.3305	0.2918	0.3743					
Age	0.02949	< .001	1.0299	1.0224	1.0375					
Number of chronic Illnesses	0.18534	< .001	1.2036	1.1606	1.2483					
Instrumental activities of daily life	0.00437	0.800	1.0044	0.9710	1.0389					
Sex: Female – Male	-0.44572	< .001	0.6404	0.5718	0.7171					
**Model comparison: 1-2**								233	4	< .001
Note. Estimates represent the log odds of "Drugs for: other heart diseases = Selected" vs. "Drugs for: other heart diseases = Not selected"										

Supplement Table S11. Binomial Logistic Regression: Drugs for: other pain

**Predictor**	**Estimate**	**p**	**Odds ratio**	**95% CI Lower**	**95% CI Upper**	**Model Fit Measures**		**Overall Model Test**		
						**R^2_N**	**AIC**	**χ^2**	**df**	**p**
**Model 1**										
Intercept	-1.3327	< .001	0.402	0.230	0.303	0.0613	10533	437	3	< .001
Depression	0.1281	< .001	1.047	1.114	1.160					
Memory	-0.0226	0.059	0.961	0.955	1.001					
Polypharmacy No – Yes	-0.6119	< .001	0.242	0.490	0.600					
**Model 2**										
Intercept	-0.3255	0.230	0.722	0.425	1.229	0.0926	10312	667	7	< .001
Depression	0.0723	< .001	1.075	1.052	1.099					
Memory	-0.0380	0.004	0.963	0.938	0.988					
Polypharmacy No - Yes	-0.4037	< .001	0.668	0.595	0.750					
Age	-0.0226	< .001	0.978	0.971	0.984					
Number of chronic Illnesses	0.1399	< .001	1.150	1.112	1.190					
Instrumental activities of daily life	0.1121	< .001	1.119	1.084	1.154					

Sex: Female – Male	0.4680	< .001	1.597	1.438	1.773					
**Model comparison: 1-2**								230	4	< .001
Note. Estimates represent the log odds of "Drugs for: other pain = Selected" vs. "Drugs for: other pain = Not selected"										

Supplement Table S12. Binomial Logistic Regression: Drugs for: osteoporosis

**Predictor**	**Estimate**	**p**	**Odds ratio**	**95% CI Lower**	**95% CI Upper**	**Model Fit Measures**		**Overall Model Test**		
						**R^2_N**	**AIC**	**χ^2**	**df**	**p**
**Model 1**										
Intercept	-2.1468	< .001	0.117	0.0964	0.142	0.0255	6282	164	3	< .001
Depression	0.0627	< .001	1.065	1.0348	1.095					
Memory	0.0118	0.482	1.012	0.9791	1.046					
Polypharmacy No – Yes	-0.7614	< .001	0.467	0.4054	0.538					
**Model 2**										
Intercept	-4.55142	< .001	0.0106	0.00490	0.0227	0.1076	5726	693	7	< .001
Depression	-0.02933	0.069	0.9711	0.94086	1.0023					
Memory	-1.79e−4	0.992	0.9998	0.96433	1.0366					
Polypharmacy No - Yes	-0.53467	< .001	0.5859	0.49782	0.6895					
Age	0.00885	0.058	1.0089	0.99972	1.0182					
Number of chronic Illnesses	0.17605	< .001	1.1925	1.13839	1.2492					
Instrumental activities of daily life	0.01853	0.416	1.0187	0.97424	1.0652					
Sex: Female – Male	1.92316	< .001	6.8426	5.52327	8.4770					
**Model comparison: 1-2**								528	4	< .001
Note. Estimates represent the log odds of "Drugs for: osteoporosis = Selected" vs. "Drugs for: other osteoporosis = Not selected"										

Supplement Table S13. Binomial Logistic Regression: Drugs for: suppressing inflammation (only glucocorticoids or steroids)

**Predictor**	**Estimate**	**p**	**Odds ratio**	**95% CI Lower**	**95% CI Upper**	**Model Fit Measures**		**Overall Model Test**		
						**R^2_N**	**AIC**	**χ^2**	**df**	**p**
**Model 1**										
Intercept	-3.2868	< .001	0.0374	0.0284	0.0492	0.0482	3673	156	3	< .001
Depression	0.1510	< .001	1.1630	1.1200	1.2076					
Memory	0.0267	0.256	1.0271	0.9808	1.0755					
Polypharmacy No – Yes	-0.7493	< .001	0.4727	0.3874	0.5768					
**Model 2**										
Intercept	-3.40875	< .001	0.0331	0.0117	0.0933	0.0526	3696	170	7	< .001

Depression	0.12201	< .001	1.1298	1.0843	1.1771					
Memory	0.02816	0.271	1.0286	0.9783	1.0815					
Polypharmacy No - Yes	-0.62256	< .001	0.5366	0.4294	0.6705					
Age	-0.00347	0.591	0.9965	0.9840	1.0092					
Number of chronic Illnesses	0.07560	0.017	1.0785	1.0139	1.1473					
Instrumental activities of daily life	0.04135	0.144	1.0422	0.9859	1.1017					
Sex: Female – Male	0.24586	0.020	1.2787	1.0400	1.5722					
**Model comparison: 1-2**								14.5	4	0.006
Note. Estimates represent the log odds of " Drugs for: suppressing inflammation = Selected" vs. " Drugs for: suppressing inflammation = Not selected"										

Supplement Table S14. Binomial Logistic Regression: Drugs for: other

Predictor	Estimate	p	Odds ratio	95% CI Lower	95% CI Upper	Model Fit Measures		Overall Model Test		
						R^2_N	AIC	χ^2	df	p
**Model 1**										
Intercept	-0.82309	< .001	0.439	0.388	0.497	0.00902	13042	69.8	3	< .001
Depression	0.01675	0.078	1.017	0.998	1.036					
Memory	0.00972	0.349	1.010	0.989	1.031					
Polypharmacy No – Yes	-0.35052	< .001	0.704	0.643	0.772					
**Model 2**										
Intercept	-1.79679	< .001	0.166	0.103	0.268	0.11128	12228	893.4	8	< .001
Depression	0.00269	0.802	1.003	0.982	1.024					
Memory	0.02585	0.026	1.026	1.003	1.050					
Polypharmacy No - Yes	-0.31771	< .001	0.728	0.654	0.810					
Age	0.01068	< .001	1.011	1.005	1.017					
Number of chronic Illnesses	-0.07719	< .001	0.926	0.896	0.957					
Instrumental activities of daily life	0.07350	< .001	1.076	1.044	1.110					
Sex: Female – Male	-0.09103	0.049	0.913	0.834	1.000					
Other: Selected – Not selected	1.47377	< .001	4.366	3.938	4.840					
**Model comparison: 1-2**								824	5	< .001
Note. Estimates represent the log odds of " Drugs for: other = Selected" vs. " Drugs for: other = Not selected"										

Supplement Table S15. Binominal Logistic regression: Drug for: Hypertension (after exclusion of people with Alzheimer´s disease, dementia, senility)

Predictor	Estimate	p	Odds ratio	95% CI Lower	95% CI Upper	Model Fit Measures		Overall Model Test		
						R^2_N	AIC	χ^2	df	p
**Model 1**										
Intercept	1.4547	< .001	4.283	3.771	4.866	0.0573	14043	470	3	< .001
Depression	-0.0439	< .001	0.957	0.940	0.975					
Memory	-0.0312	0.002	0.969	0.951	0.988					
Polypharmacy No – Yes	-0.9938	< .001	0.370	0.336	0.408					
**Model 2**										
Intercept	-3.1997	< .001	0.0408	0.0202	0.0821	0.7234	6216	8278	8	< .001
Depression	-0.0182	0.265	0.9819	0.9510	1.0139					

Memory	0.0173	0.317	1.0174	0.9836	1.0524			
Polypharmacy No - Yes	-1.0828	< .001	0.3387	0.2880	0.3983			
Age	0.0393	< .001	1.0401	1.0313	1.0490			
Number of chronic Illnesses	-0.1489	< .001	0.8617	0.8143	0.9119			
Instrumental activities of daily life	-0.0253	0.320	0.9750	0.9276	1.0248			
Sex: Female – Male	-0.1488	0.031	0.8617	0.7529	0.9863			
Hypertension: Selected – Not selected	5.0630	< .001	158.0667	131.6839	189.7353			
**Model comparison: 1-2**						7808	5	< .001
Note. Estimates represent the log odds of " Drugs for: hypertension = Selected" vs. " Drugs for: hypertension = Not selected"								

Supplement Table S16. Mediation model for Drugs for: diabetes

**Indirect and Total Effects**								
				**95% C.I. (a)**				
**Type**	**Effect**	**Estimate**	**SE**	**Lower**	**Upper**	**β**	**z**	**p**
Indirect	Polypharmacy ⇒ depression ⇒ Drugs for diabetes	0.00378	0.00101	0.00184	0.00580	0.00463	3.751	< .001
	Polypharmacy ⇒ memory ⇒ Drugs for diabetes	-0.00129	5.94e-4	-0.00244	-1.09e−4	-0.00158	-2.175	0.030
	age ⇒ depression ⇒ Drugs for diabetes	-1.80e−5	1.88e-5	-5.54e−5	1.83e-5	-3.96e−4	-0.958	0.338
	age ⇒ memory ⇒ Drugs for diabetes	8.81e-4	1.23e-4	6.39e-4	0.00112	0.01938	7.151	< .001
	sex ⇒ depression ⇒ Drugs for diabetes	-0.00535	0.00136	-0.00806	-0.00273	-0.00698	-3.931	< .001
	sex ⇒ memory ⇒ Drugs for diabetes	-0.00341	6.48e-4	-0.00466	-0.00212	-0.00445	-5.261	< .001
	No of chronic illnesses ⇒ depression ⇒ Drugs for diabetes	-0.00173	4.56e-4	-0.00265	-8.60e−4	-0.00724	-3.803	< .001
	No of chronic illnesses ⇒ memory ⇒ Drugs for diabetes	7.31e-4	1.96e-4	3.43e-4	0.00111	0.00305	3.724	< .001
	IADL ⇒ depression ⇒ Drugs for diabetes	-0.00280	7.23e-4	-0.00425	-0.00141	-0.01159	-3.871	< .001
	IADL ⇒ memory ⇒ Drugs for diabetes	0.00316	4.50e-4	0.00228	0.00404	0.01310	7.036	< .001
Component	Polypharmacy ⇒ depression	-0.55277	0.05223	-0.65471	-0.44999	-0.10870	-10.584	< .001
	depression ⇒ Drugs for diabetes	-0.00685	0.00173	-0.01031	-0.00352	-0.04263	-3.951	< .001
	Polypharmacy ⇒ memory	0.10570	0.04596	0.01438	0.19453	0.02300	2.300	0.021

	memory ⇒ Drugs for diabetes	-0.01222	0.00166	-0.01545	-0.00893	-0.06876	-7.354	< .001
	age ⇒ depression	0.00263	0.00262	-0.00245	0.00781	0.00929	1.005	0.315
	age ⇒ memory	-0.07206	0.00223	-0.07656	-0.06783	-0.28183	-32.360	< .001
	sex ⇒ depression	0.78163	0.03765	0.70636	0.85394	0.16374	20.761	< .001
	sex ⇒ memory	0.27912	0.03577	0.20827	0.34847	0.06472	7.804	< .001
	No of chronic illnesses ⇒ depression	0.25341	0.01588	0.22239	0.28463	0.16994	15.959	< .001
	No of chronic illnesses ⇒ memory	-0.05983	0.01346	-0.08612	-0.03335	-0.04441	-4.444	< .001
	IADL ⇒ depression	0.40911	0.01666	0.37663	0.44193	0.27199	24.558	< .001
	IADL ⇒ memory	-0.25888	0.01138	-0.28127	-0.23664	-0.19049	-22.740	< .001
Direct	Polypharmacy ⇒ Drugs for diabetes	-0.11861	0.00984	-0.13791	-0.09934	-0.14523	-12.053	< .001
	age ⇒ Drugs for diabetes	-0.00214	4.36e-4	-0.00298	-0.00127	-0.04712	-4.909	< .001
	sex ⇒ Drugs for diabetes	-0.04396	0.00717	-0.05786	-0.02974	-0.05734	-6.128	< .001
	No of chronic illnesses ⇒ Drugs for diabetes	0.06630	0.00289	0.06058	0.07191	0.27688	22.930	< .001
	IADL ⇒ Drugs for diabetes	-0.00553	0.00296	-0.01142	1.71e-4	-0.02290	-1.871	0.061
Total	Polypharmacy ⇒ Drugs for diabetes	-0.11612	0.00836	-0.13249	-0.09974	-0.14223	-13.897	< .001
	age ⇒ Drugs for diabetes	-0.00128	4.33e-4	-0.00213	-4.29e−4	-0.02814	-2.950	0.003
	sex ⇒ Drugs for diabetes	-0.05272	0.00682	-0.06608	-0.03936	-0.06880	-7.735	< .001
	No of chronic illnesses ⇒ Drugs for diabetes	0.06530	0.00245	0.06049	0.07011	0.27279	26.629	< .001
	IADL ⇒ Drugs for diabetes	-0.00517	0.00239	-0.00985	-4.87e−4	-0.02140	-2.164	0.030
Note. Confidence intervals computed with method: Parametric bootstrap								
Note. Betas are completely standardized effect sizes								
Note: IADL = instrumental activities of daily living								
